# Living conditions and quality of care in residential units for people with long-term mental illness in Portugal – a cross-sectional study

**DOI:** 10.1186/s12888-016-0743-7

**Published:** 2016-02-20

**Authors:** Graça Cardoso, Ana Papoila, Gina Tomé, Helen Killaspy, Michael King, José Miguel Caldas-de-Almeida

**Affiliations:** Chronic Diseases Research Center (CEDOC), NOVA Medical School/Faculdade de Ciências Médicas, Universidade Nova de Lisboa, Campo dos Mártires da Pátria 130, 1169-056 Lisboa, Portugal; NOVA Medical School/Faculdade de Ciências Médicas, Universidade Nova de Lisboa, Campo dos Mártires da Pátria 130, 1169-056 Lisboa, Portugal; Division of Psychiatry, University College London, 6th Floor, Maple House, 149 Tottenham Court Road,, London, W1T 7NF UK

**Keywords:** Long-term, Mental illness, Quality of care, Residential units

## Abstract

**Background:**

As in most European countries, mental health care has shifted from large hospitals to smaller community based settings in Portugal. Our study objectives were to determine: a) the characteristics of users of mental health residential facilities in Portugal; b) the quality of care provided comparing community and hospital units; and c) to investigate associations between quality of care, service and service users’ characteristics and experiences of care.

**Methods:**

All longer term mental health units in Portugal providing on-site staffed support for at least 12 h per day were assessed with the Quality Indicator for Rehabilitative Care (QuIRC), a standardised tool completed by the unit manager. The QuIRC rates seven domains of care (Living Environment, Therapeutic Environment, Treatments and Interventions, Self/Management and Autonomy, Recovery Based Practice, Social Inclusion, and Human Rights). A random sample of service users were interviewed using standardised measures of autonomy, experiences of care and quality of life.

**Results:**

Most (60 %) of the 42 units were in Lisbon and surrounding districts with 50 % based in the community and 50 % in hospital settings. They had a mean of 11.5 beds. Service users (*n* = 278) were mainly men (66.2 %), with a diagnosis of schizophrenia (72.7 %), and a mean age of 49.4 years. Community units scored higher than hospital units on the Living Environment, Treatments and Interventions, and Self-Management and Autonomy domains of the QuIRC. Increased service user age was negatively associated with all but one domain. All QuIRC domains were positively associated with service users’ autonomy and experiences of care.

**Conclusions:**

Investing in better quality, community based mental health facilities is associated with better outcomes for service users who require longer term support.

## Background

Following recommendations from the European Commission’s Green Paper [1], most European countries have made significant advances in the development of community based mental health services [2]. An increasing number of patients who previously spent many years in psychiatric hospitals can now live in the community, either independently or with a variable degree of support [2].

The development of community services for the treatment and rehabilitation of people with longer term mental health problems is currently a priority across Europe [3]. This includes appropriately supported accommodation in urban residential areas.

Longitudinal studies have shown encouraging results for most people diagnosed with mental health problems such as schizophrenia [4]. Even patients with more complex problems and high levels of need have been shown to have generally good outcomes [5]. Moving from a psychiatric institution to community-based care has been associated with improvements in psychological, physical and social health and wellbeing [6, 7].

In Portugal, community based mental health services were developed since 1998. In 2006, 220 people with severe, longer term mental health problems were living in residential units in the community across the country, while almost 5000 were still living in psychiatric institutions, and many others were waiting for a vacancy in a residential unit [8]. The introduction of legislation [9] [8] and approval of the first National Mental Health Plan [10], spurred the development of community services for people with severe mental illness, and in particular patients with schizophrenia and schizoaffective disorders. Moreover, the National Mental Health Plan [10] includes specific strategies to develop comprehensive services for people with severe mental illness or psychosocial disability, particularly for those who cannot live independently, with a major focus on housing.

Additionally, the recent National Program for Continuing Care in Mental Health [11] has provided a unique opportunity to develop a greater number of residential units and highlighted the need for assessing the quality of care they provide, and the impact that treatment and rehabilitation programs can have on those who use these services. Until recently, there were no reliable tools for assessing quality of care in residential facilities for people with severe mental illness. The Quality Indicator for Rehabilitative Care (QuIRC) is an instrument developed in the multinational study “Development of a European Measure of Best Practice for People with Long Term Mental Illness in Institutional Care (DEMoBinc)” funded by the European Commission, that aims to fill this gap [12–14].

The main objectives of this study were to determine: a) the characteristics of users of mental health residential facilities in Portugal; b) the quality of care provided in general and comparing community and hospital units; and c) to investigate associations between quality of care, service and service users’ characteristics and experiences of care. This study was made possible by funding from the Ministry of Health, and was approved by the Ethics Committee of the NOVA University of Lisbon Medical School.

## Methods

### Data collection

All the Portuguese residential units for people with longer term mental health problems with high or medium support levels (i.e., at least 12 h on-site staff support per day) were contacted and invited to participate in the study. Service managers were sent written information about the study and given the opportunity to ask questions about it. Written informed consent for their participation was gained. Units that provided specialist care (for example only for people with dementia, severe cognitive impairment or learning disability) and units with fewer than six residents were excluded. Data collection took place between March and July 2012.

### Instruments

Each unit that accepted to participate was assessed with the Portuguese version of the QuIRC, a web-based toolkit completed online by the unit manager (available at www.quirc.eu). This instrument assesses the quality of care of longer-term units for people with complex mental health problems on seven domains of care (Living Environment; Therapeutic Environment; Treatments and Interventions; Self-Management and Autonomy; Recovery Based Practice; Social Inclusion; Human Rights). Its content was derived from a systematic literature review of the effectiveness of components of care for this group [15], a review of relevant international care standards, and Delphi exercises with service users, practitioners, carers and advocates [16]. The QuIRC has excellent inter-rater reliability and good internal validity (Killaspy et al, 2012) [14]. It takes about 45 min to complete and comprises 145 questions about service provision (e.g. number of beds, average length of stay, built environment, treatments and interventions, staffing, staff turnover, training, and supervision); links with community organizations (e.g. colleges, employment agencies, sport and leisure facilities); the therapeutic milieu and recovery-based practices (e.g. collaborative care planning, service user involvement, promotion of service users independent living skills); and the protection of service users’ human rights (e.g. their privacy and dignity, their legal rights and the use of restraint and seclusion). Domain scores are calculated from scores on 86 items and range from 0 to 100 %, with higher scores meaning better quality of care. The remaining items provide descriptive data.

Additional descriptive data on the users’ sociodemographic characteristics, psychiatric diagnosis, psychotropic drugs taken, and length of stay in the unit were provided by the unit staff in advance of users interviews.

In units with between 6 and 10 beds all the service users were invited to participate, while in larger units a randomized sample of ten service users was approached. Service users who gave their written informed consent participated in a face-to-face research interview taking about 30 min. Autonomy was assessed using the Resident Choice Scale (RCS) [17]; the service user rates the degree to which they have choice over 22 aspects of daily activities and the running of the unit on a four-point scale, with total scores ranging from 22 to 88. Quality of life was assessed using the Manchester Short Assessment of Quality of Life (MANSA) [18]; the service user rates 12 aspects of their life on a scale from 1 (couldn’t be worse) to 7 (couldn’t be better), with a generated mean score ranging from 1 to 7. The users’ experiences of care were assessed using the Your Treatment and Care (YTC) questionnaire [19], which includes 25 items related to a person’s care that are noted as being present or not, thus providing a total score between 0 and 25. Service users’ views on the unit’s therapeutic milieu were assessed using the General Milieu Index (GMI) [20] which comprises four items rated between 1 and 5, providing a total score between 4 and 20. Service user functioning was also assessed by the interviewer using the Global Assessment of Functioning (GAF) [21] in order to take this into account as a potential moderator of the relationship between service quality and clinical outcomes. The GAF is widely used, and part of the Diagnostic and Statistical Manual (DSM) of Mental Disorders [22]. The researcher rates the person’s overall symptoms and functioning on a scale between 1 and 100, with lower scores showing greater disability.

The user interviews were conducted by experienced psychiatrists and psychologists trained for the purpose. All service user interviews were completed within 2.1 (SD = 1.1) months of the manager’s assessment of the unit.

### Statistical analysis

Descriptive data are presented as frequencies and percentages, means and standard deviations (SD) or medians, ranges (R), and interquartile ranges (IQR), as appropriate. Student’s *t*-test or Mann–Whitney test were used for quantitative variables whenever needed.

**Fig. 1:**
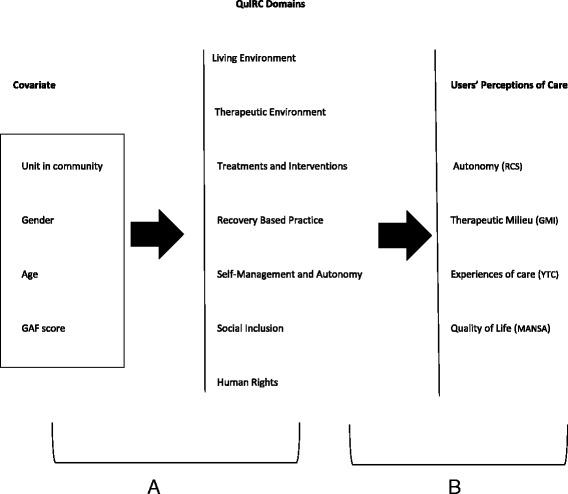
Diagram of the studied associations

Univariable and multivariable linear regression models were used to investigate which covariates were associated with unit quality (QuIRC domain scores) (Fig. 1a). Covariates considered a priori were: location of unit (hospital or community); percentage of male service users; mean age of service users; and service users’ mean GAF score.

In order to investigate whether unit quality (QuIRC domain scores) was associated with service user perceptions of care and the care environment, namely autonomy (RCS), quality of life (MANSA), experiences of care (YTC) and therapeutic milieu (GMI), mixed effects models were used (Fig. 1b). These hierarchical models take into account the correlation structure between patients clustered in units. As YTC data were highly asymmetric, they were dichotomized (below and above 18, the median score). The level of significance α = 0.05 was considered. Data were analysed using Stata (StataCorp. 2011. Stata Statistical Software: Release 12. College Station, TX: StataCorp LP).

## Results

The total number of units meeting the inclusion criteria in Portugal was 42. All were invited to participate, and all accepted the invitation. The units were distributed across the country, although the majority (*n* = 25, 59.5 %) was in the Greater Lisbon Region.

The total number of beds occupied in the 42 units was 499, the majority by men (*n* = 327, 65.5 %). The number of users invited to participate in the study was 355 (due to the randomized choice in units with more than 10 users). Forty-three users declined participation, 13 were absent, and 21 lacked capacity to be interviewed, leaving 278 (78.3 %) who participated in research interviews. In community facilities it was more frequent that users were not available for the interview because of other activities, while in the hospital units it was more frequent for users to decline participation or to have high cognitive impairment preventing interview. Thus 42 units and 278 service users were included in the analysis. No missing values were present in our data.

### Unit characteristics

As shown in Table 1, more than half of the units (54.8 %) were based in the community rather than hospital settings and there was an even spread across urban, suburban and rural areas. Twenty-three units were single sex only (54.8 %), with 14 being male only (33.3 %), and 9 female only (21.4 %).

**Table 1:** Characteristics of the residential units

	N (%)	Mean (SD)	No access	Access outside unit	Works in unit
Unit location^a^					
Inner city	16 (38.1)				
Suburbs	11 (26.2)				
Countryside	15 (35.7)				
Unit type^a^					
In hospital context	19 (45.2)				
In the community	23 (54.8)				
Staffing^a^					
Psychiatrist			2 (4.8)	20 (47.6)	20 (47.6)
Psychologist			0 (0.0)	16 (38.1)	26 (61.9)
Occupational Therapist			11(26.2)	10 (23.8)	21 (50.0)
Nurse			2 (4.8)	13 (31.0)	27 (64.2)
Auxiliary			5 (11.9)	4 (9.5)	33 (78.6)
Social Worker			0 (0.0)	10 (23.8)	32 (76.2)
Counsellor/Psychotherapist			25 (59.5)	14 (33.3)	3 (7.1)
Art Therapy			19 (45.2)	18 (42.9)	5 (11.9)
Beds available	524	12.5 (8.0)			
Beds occupied	499	11.9 (7.1)			
% beds occupied		96.2 (6.5)			

^a^ *N* = 42

The majority of units had at least 10 beds (61.9 %) and no maximum length of stay (88.1 %). No service users were involuntarily detained.

More than half of the units had no single bedrooms (*n* = 22, 52.8 %), and in 16 units (38 %), more than two service users shared a bedroom, with eight people sharing a room in one unit. Three units included patients who were under community treatment orders but these made up only four users in all.

The current model of care had been used in the units on average for 9.1 (SD = 5.5) years, ranging from 1 to 22 years.

#### Staffing

All units provided access to psychologists and social workers, and most (95 %) had access to a psychiatrist and nurses (95.2 %), nursing auxiliaries (88.1 %), and occupational therapists (73.8 %). Less than half provided access to psychotherapy.

Most units were staffed 24 h a day (54.8 %), and the remainder provided staffing out of usual working hours only, because service users were attending day programs outside the unit during the daytime.

The median staff per unit was 14 (IQR 10–15.5), with 7 (IQR 4–13) being on permanent contracts. The median full time equivalent (FTE) staff per unit was 2.4 [R 0–20.7]. The mean staff turnover in the last two years was 17.1 %. Only one unit employed an ex-service user as a member of staff.

#### Staff training and activities carried out

Most of the units had provided staff training in the last 12 months in service users’ rights (73.8 %), mental health law (61.9 %), communication skills (64.3 %), recovery based practice (54.8 %), and talking therapies (52.4 %). Fewer units had provided training in working with families (47.6 %), health promotion (38.1 %), smoking cessation (21.4 %), de-escalation techniques and physical restraint (17.7 % each).

Individualised care plans were used in almost all (90.7 %) units, while individualised activities programs only existed in 28 (66.7 %). Regular organized activities inside the units existed in 40 (95.2 %) of units. Thirty-five units (83.3 %) offered a “keyworker” to their users.

The managers reported various approaches to helping service users access community activities, including developing links with local entertainment resources such as cinemas (69.0 %), churches/religious associations (64.3 %), coffeeshops/restaurants (59.5 %), and sports facilities (59.5 %).

#### Units’ objectives

Half the unit managers reported that the service’s main objective was to help users to live more independently. However, 17 (40.5 %) managers also considered providing care for people with disability as an equal priority. In 14 (33.3 %) units, managers reported that more than half of their service users could do most things without help, while 25 (59.5 %) reported that most of their service users could do very little without assistance from staff. Twenty-three (54.8 %) managers said that a mean of 14.7 % of their users had moved to more independent living in the last two years. Most unit managers (*n* = 31, 73.8 %) believed that the majority of their service users would improve in their day to day functioning over the next two years, but only 11 (26.2 %) expected at least a quarter of them to move on to more independent accommodation.

#### Psychotropic medication

According to the managers’ information, from the total of 499 service users 295 (59.1 %) were prescribed atypical anti-psychotics and 263 (52.7 %) were prescribed typical antipsychotics. A quarter (121, 24.2 %) were prescribed clozapine. Almost half (244, 48.9 %) were prescribed more than two anti-psychotics.

#### QuIRC dimensions

Table 2 shows the results of the quality of care as assessed by the QuIRC. The mean scores were above 50 % in most dimensions except Therapeutic Environment and Recovery Based Practice. Quality of care was higher in community based units compared to hospital units in the following domains: Living Environment (*p* = 0.030), Treatments and Interventions (*p* = 0.035), and Self-Management and Autonomy (*p* = 0.019). The Portuguese units’ mean scores on the QuIRC domains were similar to those from across Europe with the exception of Therapeutic Environment (47.1 for Portugal *vs* 52.1 for Europe, *p* = 0.005), and Recovery Based Practice (44.9 *vs* 57.0, *p* < 0.001) (unpublished data available from Helen Killaspy).

**Table 2:** Comparison of QuIRC domains scores in Portuguese community and hospital units, mean (SD) minimum-maximum. Scores from England and across Europe are also shown for reference

QuIRC Domains	Portuguese Units	Community Units	Hospital Units	p*	England units	Europe units
	*N* = 42	*n* = 23	*n* = 19		*n* = 20	*n* = 213
Living Environment	63.8 (11.4)	67.2 (10.5)	59.7 (11.3)	**0.030**	67.0 (10.7)	59.8 (15.5)
	41.8–91.0	43.4–91.0	41.8–79.5		48.4–86.1	15.6–89.3
Therapeutic Environment	47.1 (11.1)	48.8 (12.4)	45.1 (9.2)	0.295	64.5 (6.0)	52.1 (9.5)
	25.2–73.0	25.2–73.0	25.3–58.5		58.1–78.4	21.0–78.4
Treatments and Interventions	50.1 (13.1)	54.0 (13.3)	45.5 (11.5)	**0.035**	59.5 (8.0)	50.6 (9.1)
	21.9–78.8	21.9–78.8	23.5–59.0		45.8–77.3	28.2–79.6
Self Management and Autonomy	54.5 (14.7)	59.2 (15.6)	48.7 (11.4)	**0.019**	68.7 (11.0)	55.5 (15.5)
	19.1–84.7	19.2–84.7	24.5–64.9		44.7–86.0	16.6–86.0
Social Inclusion	52.0 (13.5)	54.8 (14.2)	48.6 (12.0)	0.135	53.9 (12.7)	56.7 (12.7)
	21.6–84.0	21.6–83.9	22.8–61.5		34.9–76.3	25.2–82.8
Human Rights	54.9 (11.7)	56.7 (12.6)	52.5 (10.4)	0.215	69.6 (9.2)	52.7 (12.7)
	33.1–77.1	33.1–77.1	35.3–69.2		51.1–82.8	15.7–81.6
Recovery Based Practice	44.9 (13.6)	46.9 (14.9)	42.4 (11.8)	0.290	65.9 (9.7)	57.0 (15.5)
	18.0–73.0	18.0–73.0	19.8–59.7		49.0–81.6	15.6–89.3

*Student’s *t*-test *p* value; p-values with statistical significance in bold

### Service user characteristics

As shown on Table 3, the 278 service users assessed were mainly male (66.2 %), with a mean age of 50.5 years (SD = 11) [R 23–83], and they had been living in the unit for a median of 4 years (IQR 1–10) [R 0–60]. The majority (73.7 %) had a diagnosis of schizophrenia, and no regular occupation (92.8 %). Most had their own bank account (55.6 %) but only 33.8 % were in charge of their finances. A quarter (25.7 %) had voted in the last election.

**Table 3:** Service user characteristics, *n* = 278

	Mean (SD) [range]	N (%)
Age, years	50.5 (11.0) [23–83]	
Male gender		184 (66.2)
Diagnosis		
Schizophrenia		205 (73.7)
Affective disorders		28 (10.1)
Intelectual disability		25 (9.0)
Personality Disorders		5 (1.8)
Substance use related disorders		3 (1.1)
Other diagnoses		12 (4.3)
Standardised outcome measures		
Autonomy (Resident Choice Scale)	57.3 (9.5) [32–87]	
Quality of Life (Manchester Short	4.8 (0.9) [2.3–6.8]	
Assessment of Quality of Life)		
Experiences of Care (Your Treatment and Care)	17.9 (4.6) [4–25]	
Therapeutic Milieu (General Milieu Index)	18.5 (4.6) [5–31]	
Social Functioning (Global Assessment of Functioning)	64.3 (15.1) [25–95]	

Service users’ ratings of their autonomy (RCS), quality of life (MANSA), experiences of care (YTC) and therapeutic milieu (GMI) of the unit are shown in Table 3. The researchers’ mean ratings of service user functioning (GAF) did not differ between community (63.5, SD = 14.9) and hospital (65.1, SD = 15.3) units (*p* = 0.389).

### Factors associated with unit quality

As shown in the univariable regression analysis (Table 4) units that were based in the community had higher quality scores on all domains of the QuIRC than hospital units, but this only reached statistical significance for Living Environment (coefficient estimate 7.6, 95 % CI 0.76 to 14.41, *p* = 0.030), Treatments and Interventions (coefficient estimate 8.5, 95 % CI 0.65 to 16.32, *p* = 0.035) and Self-Management and Autonomy (coefficient estimate 10.5, 95 % CI 1.80 to 19.19, *p* = 0.019).

**Table 4:** Association between unit quality (QuIRC domain scores), unit location, and service user characteristics

	Coefficient estimate	95 % CI	*p*
Living Environment			
Unit in community	7.6	(0.76 to 14.41)	0.030
Male%^a^	-0.9	(-1.81 to -0.011)	0.047
Mean age, years^b^	-2.9	(-5.46 to -0.28)	0.031
GAF score^c^	4.6	(1.26 to 7.89)	0.008
Therapeutic Environment			
Unit in community	3.7	(-3.31 to -10.61)	0.295
Male%^a^	-0.3	(-1.17 to -0.67)	0.582
Mean age, years^b^	-2.5	(-5.08 to -0.02)	0.052
GAF score^c^	1.1	(-2.44 to -4.59)	0.539
Treatments and Interventions			
Unit in community	8.5	(0.65 to 16.32)	0.035
Male%^a^	-0.2	(-1.23 to 0.93)	0.774
Mean age, years^b^	-4.6	(-7.36 to -1.80)	0.002
GAF score^c^	2.1	(-1.94 to 6.23)	0.295
Recovery Based Practice			
Unit in community	4.5	(-4.00 to 13.05)	0.290
Male%^a^	-0.1	(-1.26 to 1.00)	0.816
Mean age, years^b^	-4.6	(-6.77 to -0.65)	0.019
GAF score^c^	2.6	(-1.60 to 6.88)	0.216
Self-Management and Autonomy			
Unit in community	10.5	(1.80 to 19.19)	0.019
Male%^a^	-0.8	(-2.01 to -0.36)	0.169
Mean age, years^b^	-4.3	(-7.58 to -1.08)	0.010
GAF score^c^	4.5	(0.05 to 8.91)	0.048
Social Inclusion			
Unit in community	6.3	(-2.03 to 14.58)	0.135
Male%^a^	0.1	(-1.04 to 1.19)	0.896
Mean age, years^b^	-4.6	(-7.46 to 1.67)	0.003
GAF score^c^	1.4	(-2.88 to 5.62)	0.519
Human Rights			
Unit in community	4.5	(-2.75 to 11.84)	0.215
Male%^a^	-0.7	(-1.63 to 0.26)	0.151
Mean age, years^b^	-1.8	(-4.6 to 0.96)	0.195
GAF score^c^	2.8	(-0.84 to 6.39)	0.128

^a^For each 10 % increase of male users

^b^For each 5-year increase

^c^For each 10-point increase

Service user characteristics were also associated with unit quality. For example, service users’ age was negatively associated with five of the seven QuIRC domains (e.g. for each 5 year increase in age there was a mean decrease of 4.6 % in Treatments and Interventions, 95 % CI -7.36 to -1.80, *p* = 0.002). The level of disability of service users was positively associated with Living Environment and Self-Management and Autonomy domain scores; an increase (towards less disability) in service users’ mean GAF score of 10 points was associated with a mean increase of 4.6 % in the Living Environment score (95 % CI 1.26 to 7.89, *p* = 0.008), and of 4.5 % in Self-Management and Autonomy (95 % CI 0.05 to 8.91, *p* = 0.048).

In the multivariable regression analysis the Living Environment mean domain score was negatively associated with the proportion of male users (coefficient estimate -0.1, 95 % CI -1.83 to -0.20, *p* = 0.016), and positively associated with service user functioning, with a mean increase of 4.9 % for each 10-points increase in the mean GAF score (95 % CI 1.78 to 8.03, *p* = 0.003). The Self-Management & Autonomy domain score was negatively associated with the proportion of male users (coefficient estimate -1.2, 95 % CI -2.32 to -0.13, *p* = 0.029), and with age, with a mean decrease of 5.1 % with every a 5-year increase in the mean age of service users’ age (95 % CI -8.32 to -1.96, *p* = 0.002).

### Unit quality and service users’ perceptions

According to univariable regression analysis shown in Table 5, all QuIRC domain scores were positively associated with service user ratings of autonomy (RCS), therapeutic milieu (GMI), and experiences of care (YTC). For example, for every increase of 10 % in the Living Environment domain score there was an increase of 2.99 (95 % CI 1.73–4.24) on the RCS scale and 44 % (OR 1.44; 95 % CI 1.09–1.89) in the odds of having the YTC scale score above the median. However, only the Therapeutic Environment domain score was positively associated with quality of life (MANSA) (coefficient estimate 1.49; 95 % CI 0.08–2.91).

**Table 5:** Association between unit quality (QuIRC domain scores) and service users’ perceptions of care

Characteristic	Odds ratio^a^ or Coefficient estimates^b^	95 % CI	*p*
Autonomy (Resident Choice Scale)^b^			
Living environment	2.99	(1.73 to 4.24)	<0.001
Therapeutic environment	2.92	(1.54 to 4.30)	<0.001
Treatments and interventions	2.75	(1.67 to 3.82)	<0.001
Self-management and autonomy	2.62	(1.71 to 3.54)	<0.001
Human rights	2.34	(1.05 to 3.64)	<0.001
Recovery-based practice	2.20	(1.02 to 3.38)	<0.001
Social inclusion	2.15	(1.05 to 3.25)	<0.001
Therapeutic milieu (General Milieu Index)^b^			
Living environment	0.77	(0.23 to 1.32)	0.006
Therapeutic environment	0.77	(0.22 to 1.33)	0.006
Treatments and interventions	0.49	(-0.003 to 0.98)	0.051
Self-management and autonomy	0.54	(0.11 to 0.96)	0.014
Human rights	0.73	(0.19 to 1.27)	0.008
Recovery-based practice	0.55	(0.08 to 1.02)	0.021
Social inclusion	0.45	(-0.02 to 0.93)	0.061
Experiences of care (Your Treatment and Care)^a^			
Living environment	1.44	(1.09 to 1.89)	0.009
Therapeutic environment	1.37	(1.03 to 1.81)	0.030
Treatments and interventions	1.29	(1.03 to 1.62)	0.027
Self-management and autonomy	1.28	(1.04 to 1.58)	0.020
Human rights	1.34	(1.02 to 1.76)	0.036
Recovery-based practice	1.27	(1.00 to 1.62)	0.053
Social inclusion	1.28	(1.02 to 1.59)	0.032
Quality of Life (MANSA)^b^			
Living environment	0.11	(-0.01 to 0.22)	0.076
Therapeutic environment	0.12	(0.01 to 0.24)	0.039
Treatments and interventions	0.06	(-0.04 to 0.16)	0.247
Self-management and autonomy	0.05	(-0.04 to 0.15)	0.259
Human rights	0.10	(-0.01 to 0.22)	0.079
Recovery-based practice	0.05	(-0.05 to 0.15)	0.344
Social inclusion	0.03	(-0.07 to 0.13)	0.610

*MANSA* Manchester Short Assessment of Quality of Life

^a^Odds ratio compares Your Treatment and Care scores below and above the median

^b^For a 10 % point change in QuIRC domain scores

## Discussion

This is the first report on residential units for people with longer term mental health problems in Portugal. A major strength of the study is the fact that all units providing high and medium levels of support in the country, as well as a large proportion of their service users, participated in the study.

The number of units had increased by 75 % (from 24 to 42) since 2009 (Killaspy et al., 2012) [14]. This is probably due to the implementation of the National Mental Health Plan (2007/2016), that has promoted a greater variety of services and more adequate care for people with severe mental illness. Most of the units were based in the community rather than hospital settings, suggesting that the process of deinstitutionalisation of mental health care in Portugal is taking place. The fact that service users’ functioning was similar in hospital and community settings suggests that this trend should continue since community based units appear able to manage people with similar levels of disability to hospital based units. Most units provided access to a multidisciplinary team and a wide range of staff training. Although it is encouraging that the quality ratings (QuIRC domain scores) were similar to the average scores across Europe, our findings suggest some important areas for improvement, especially with regard to Recovery Based Practice and Therapeutic Environment. In comparison with units in England, where the implementation of community based care has been ongoing for longer, Portuguese units scored lower on all QuIRC domains.

Although most of the units were located in urban and suburban areas, the fact that one third were in rural areas may present particular logistic difficulties in terms of access to public transport and community activities that promote social inclusion. As a comparison, in England, only 8 % of similar units are in rural areas [23]. However, this should take into account that, in 2012, Portugal’s rural population was 38 % compared to 18 % in the UK [24].

The fact that over half the units had no single bedrooms needs to be addressed since this does not comply with the Convention on the Rights of Persons with Disabilities (CRPD) [25] recommendations, such as privacy and dignity. In addition, although the number of units had increased in recent years, the model of care being used had not changed for many years in many services, suggesting that institutional practices continue. This might explain the lower scores for Therapeutic Environment and Recovery Based Practice. For example, although most services reported using individualised care plans to support service users, activity plans tailored to the individual were not usual. In addition, managers predicted that only a minority of their service users were likely to move on to more independent settings. This suggests a degree of therapeutic pessimism that requires challenging, since holding and promoting hope for a person’s recovery is a key component of recovery-based practice [26]. A further marker of recovery based practice is the employment of peer support workers in a service, yet only one unit had an ex-service user amongst their staff.

The high percentage of Portuguese service users taking more than two anti-psychotics (48.9 %) compared to those in similar units in England (3.9 %) [23] is of concern. Due to the risks involved, this issue needs to be addressed in future interventions to train the residential facilities’ staff, particularly psychiatrists. Moreover, specific Portuguese guidelines for the prescription of antipsychotics are available [27], but are clearly not being followed.

The community units in this study scored higher than hospital based units on the QuIRC domains Living Environment, Treatments and Interventions, and Self-Management and Autonomy. This finding concurs with previous studies that have found that community based units provide more homely and therapeutic environments [15]. Service users’ age was negatively associated with all but one of the QuIRC domain scores (a reduction in the score of between two and five percent for every five year increase in mean age). The level of service users’ disability was also associated with two QuIRC domains; i.e. higher mean GAF scores (more able residents), were positively associated with Living Environment and Self-Management and Autonomy. However, the gender of service users had little influence on ratings of the units’ quality.

We also found that greater quality of care on all the QuIRC domains was positively associated with service users’ autonomy, ratings of the unit’s therapeutic milieu and experiences of care. However, the only aspect of care that positively influenced service users’ quality of life was the Therapeutic Environment score. In sum, lower quality units were more likely to be situated in hospitals and contain older residents who despite having no greater disability, report lower autonomy, and rate their unit lower on therapeutic milieu. This suggests that the recent expansion in residential facilities in Portugal have superseded older, less community-focused units that have a residue of older residents. Although clearly this reform has been a positive development, the older hospital based units may need more resources to come up to European standards. This provides important evidence for continued investment to ensure higher quality of care is provided to those with longer term mental health problems.

## Conclusions

The number of residential facilities for people with longer term mental illness in Portugal has increased dramatically in the last few years. Although the quality of care provided is similar to the rest of Europe, our results provide insights into the areas where there is room for improvement. First, this type of facility should be community based in urban/suburban areas. Secondly, they should provide care to adults of different ages and with different levels of functioning. Thirdly, a greater focus on recovery-based practice is required. Finally, concerted efforts should be made in order to improve adherence to international guidelines on the prescription of antipsychotics for this group of users. Investing in the quality of care provided in these settings is likely to lead to improvements in service user autonomy and experiences of care.

## Abbreviations

RCS: Resident Choice Scale
DSM: Diagnostic and Statistical Manual of Mental Disorders
GAF: Global Assessment of Functioning
GMI: General Milieu Index
MANSA: Manchester Short Assessment of Quality of Life
QuIRC: Quality Indicator for Rehabilitative Care
YTC: Your Treatment and Care questionnaire
